# A Communication Model to Integrate the Request-Response and the Publish-Subscribe Paradigms into Ubiquitous Systems

**DOI:** 10.3390/s120607648

**Published:** 2012-06-07

**Authors:** Carlos Rodríguez-Domínguez, Kawtar Benghazi, Manuel Noguera, José Luis Garrido, María Luisa Rodríguez, Tomás Ruiz-López

**Affiliations:** Department of Computer Languages and Systems, E. T. S. I. I. T. University of Granada, C/Periodista Daniel Saucedo Aranda S/N, 18071 Granada, Spain; E-Mails: benghazi@ugr.es (K.B.); mnoguera@ugr.es (M.N.); jgarrido@ugr.es (J.L.G.); mlra@ugr.es (M.L.R.); tomruiz@ugr.es (T.R.-L.)

**Keywords:** ubiquitous systems, communication paradigms, middleware design, quality properties, abstract models

## Abstract

The Request-Response (RR) paradigm is widely used in ubiquitous systems to exchange information in a secure, reliable and timely manner. Nonetheless, there is also an emerging need for adopting the Publish-Subscribe (PubSub) paradigm in this kind of systems, due to the advantages that this paradigm offers in supporting mobility by means of asynchronous, non-blocking and one-to-many message distribution semantics for event notification. This paper analyzes the strengths and weaknesses of both the RR and PubSub paradigms to support communications in ubiquitous systems and proposes an abstract communication model in order to enable their seamless integration. Thus, developers will be focused on communication semantics and the required quality properties, rather than be concerned about specific communication mechanisms. The aim is to provide developers with abstractions intended to decrease the complexity of integrating different communication paradigms commonly needed in ubiquitous systems. The proposal has been applied to implement a middleware and a real home automation system to show its applicability and benefits.

## 1. Introduction

Ubiquitous systems are increasingly being adopted due to the growing capabilities and the commercial success of mobile devices (smartphones, netbooks, tablets, *etc.*). In these systems, entities (services, applications, agents or devices) exchange information in a shifting networking environment [1] where quality properties, like efficiency, mobility support, adaptability, reliability, security and timeliness, are required [2–5]. The fulfillment of these requirements is usually achieved by software applications and services built on top of middleware solutions, which, in turn, are mainly based on the *Request-Response* (RR) or the *Publish-Subscribe* (PubSub) paradigms. Likewise, different technologies have been proposed in order to provide specific mechanisms to support each communication paradigm [6–8].

RR is a simple paradigm to exchange information through message passing that is widely used in distributed systems. In this paradigm, a sender requests information to a receiver, which replies the required information. The message passing semantics of this paradigm have been applied as a primitive to develop more complex communication schemes (like PubSub) [9] and used in very common communication protocols, like HTTP. Different implementations of the RR paradigm have been proposed in order to take into consideration different requirements: one-way requests (the response is only a status message), batch requests (several requests codified as a single one in order to improve efficiency), RPC (requests codify a remote procedure call [10] or a method invocation [11], whereas the response is the result of its execution), *etc.* Note that RPC is the most widely used specification of the RR paradigm. Several authors have even also previously considered RPC as a paradigm itself [4,12,13].

The PubSub paradigm emulates the human procedure of subscribing to a publication: from the moment a subscriber expresses its interest in certain information, it will automatically receive a copy of the information each time it is released. From a technological perspective, it is usually implemented by designing an *event broker* (usually, as a service) that stores the subscriptions and receives all the publications [14]. When a new publication is received, this *event broker* distributes it among the subscribers. The PubSub paradigm is mainly used to notify changes in the internal state of a sender (publisher) to a set of interested receivers (subscribers). For example, in a home automation monitoring system, if a light is switched from *off* to *on*, then an event should notify applications of this occurrence, enabling the possibility of reflecting this state change in their corresponding user interfaces.

The RR paradigm is based on a one-to-one interaction model that provides a limited support for time, space and synchronization decoupling [9], which makes it not well suited for mobility support, since the coupling between senders and receivers may lead to undesirable situations. For instance, when a mobile information provider is no longer available in a system, an information requester could be indefinitely blocked while waiting for a response. However, the PubSub paradigm has proven to promote mobility support and provides efficient mechanisms for transferring one-to-many notifications [15]. Nevertheless, the decoupling between subscribers and publishers makes it difficult to guarantee a reliable delivery. Thus, software solutions for ubiquitous systems need to adopt and make simultaneous use of the RR and PubSub-based communication mechanisms available in different middleware technologies [16].

In this paper, we propose a communication model intended to integrate the PubSub and the RR paradigms and, therefore, to enable the use of the most suitable communication semantics as required, *i.e.*, on the basis of the quality properties that are usually required in ubiquitous systems. The proposal of this model is supported by the analysis of several key properties for ubiquitous systems, namely, efficiency, mobility support, adaptability, reliable delivery, security and timeliness. However, the goal of the proposal presented herein is not to directly fulfill those quality properties, but to help take them into consideration by means of design decisions, which should also be supported by implementations, based on the integration of models for specific communication paradigms, and on the basis of well-established software engineering best practices. The applicability and benefits of the proposal have been studied through the implementation of a middleware and a real home automation system.

The remainder of the paper is structured as follows: Section 2 analyzes the quality properties that the communication mechanisms implementing both the RR and the PubSub paradigms help to fulfill. Section 3 introduces a communication model that aims to take advantage of the benefits that both the PubSub and the RR paradigms provide. To show the applicability of the proposal, a home automation system developed on the basis of the proposed model and a developed middleware is described in Section 4. In Section 5 other works related to the proposal are presented. Finally, Section 6 summarizes the main contribution and additional lines of future work.

## 2. Analysis of Quality Properties and Communication Paradigms

In ubiquitous systems, it is very common to establish communication schemes based on either the PubSub or RR paradigms. Each communication paradigm provides orthogonal functionalities and promotes different quality properties, however most existing ubiquitous systems actually need to fulfill a combination of the functional and non-functional requirements fostered by each paradigm. For example, in a home automation environment, it is usually required to directly interact with specific devices through well-known interfaces or through message passing, thus being appropriate to choose RR-based communications. On the other hand, when a device changes its state (a door is opened, for instance), the applications should be notified, so as to update their GUI. In this case, the use of PubSub-based communications is more suitable.

In this section, we analyze how each communication paradigm helps to promote certain quality properties, such as efficiency, mobility support, adaptability, reliable delivery and timeliness. We also analyze the limitations of the PubSub and RR paradigms in order to highlight the need for abstractions to allow their seamless integration. These abstractions would allow avoiding *ad-hoc* solutions that simultaneously make use of different middleware technologies, each one of them usually supporting only one communication paradigm. It is important to note that the quality properties that are mentioned in this section can be achieved with the appropriate implementations of either RR or PubSub mechanisms. The problem is the impact that they will have in other requirements and the high level of complexity needed to fulfill them. These problems will negatively affect the performance of the systems that are built on top of them. For example, a PubSub proxy could ensure reliable delivery, however, by using a proxy, all the communication will need to be centralized in it. This will avoid to use decentralized implementations of the PubSub paradigm and require replication mechanisms in order to avoid bottlenecks. However, by using the RR paradigm, reliable delivery requirements are directly met.

Table 1 outlines the contribution of each communication paradigm to the quality properties that are very often sought for ubiquitous systems [2–5].

A more detailed explanation of the information included in the previous table is described as follows:

- **Efficiency.** The detection of state changes in nearby entities (*i.e.*, other applications, services, agents or devices) is a common operation in ubiquitous systems. The RR paradigm semantics involves sending periodical messages to retrieve the state of other entities, which is known as *polling*. Polling operations are usually considered very inefficient in comparison with the scheme supported by the PubSub paradigm [17], since such changes infrequently occur and memory, CPU and power resources are wasted when sending useless messages. Moreover, in RR, to distribute information to a set of receivers, the number of messages to be sent must be equal to the number of receivers. In PubSub, publishers always distribute one message, regardless of the number of subscribers. In spite of these weaknesses, the RR paradigm can be more convenient if the notification of changes in the state of the entities is time-constrained or if power consumption must be controlled periodically. As a consequence, both the PubSub and RR paradigms may help to achieve efficiency in ubiquitous systems. The choice between the two paradigms depends on the other specific requirements for each system. For example, if a piece of information has to be delivered to a wide range of receivers and it is not possible to pre-establish when it is going to be sent, nor its delivery frequency, then the PubSub paradigm should be selected in this case in order to improve efficiency. On the contrary, if the delivery times are known a priori, and the number of recipients is small, then the RR paradigm would be more efficient.
- **Mobility Support.** The PubSub paradigm promotes the decoupling between publishers and subscribers. In particular, in PubSub-based communications, it is totally transparent if either a publisher or a subscriber is present or not in a system. In RR, if a receiver is no longer available in a system, due to the coupling between senders and receivers, the execution flow of a sender could be indefinitely blocked waiting for a response that could never be received since the provider could never be present again. Additionally, the execution flow of a sender usually depends on the specific results that are extracted from the responses of the receivers. Thus, in some cases, senders may not be able to continue their execution if specific recipients are not available. Therefore, the PubSub paradigm contributes to support mobility in ubiquitous systems, whereas the RR paradigm offers no mechanisms to support it [3].
- **Adaptability.** RR-based communications require establishing well-defined interfaces to exchange messages between senders and receivers. However, in ubiquitous systems, the support to context-awareness features involves to dynamically adapt the functionality provided by services and applications to the information retrieved from the context (that is, nearby users, their tasks, available resources, *etc.*) [18]. Consequently, RR communications are not flexible enough to promote adaptability [2]. However, in PubSub communications, subscriptions may be dynamically established and dropped depending on the context. Thus, the PubSub paradigm is more suitable for building adaptable, ubiquitous systems.
- **Reliable Delivery.** Reliable delivery means that a receiver (or a set of receivers) has to send an acknowledgement for each received message in order to confirm their reception. In RR, receiving a response to a request implies that the request was delivered correctly. However, in PubSub communications, reliable delivery implies detecting from a publisher (*i.e.*, not only from the *event broker*, that is, the intermediary entity between publishers and subscribers) whether a set of subscribers have received a specific notification or not. This is only possible by reducing the decoupling between publishers and subscribers [2]. For example, in order to provide reliable delivery in the PubSub paradigm, the publishers should know, at least, the number of subscribers and an identification associated with each subscriber. Consequently, the publishers should receive an acknowledgement message from each subscriber. As a consequence, it is not possible to assume that a notification is always received when the decoupling between publishers and subscribers is a strong requirement. Thus, when reliable delivery must be ensured, RR should be used instead.
- **Security.** Security is an important concern in ubiquitous systems. Hence, the information to be exchanged should be encrypted and trusting mechanisms established for senders and receivers. Obviously, information can be encrypted in both the PubSub and the RR paradigms. However, trusting mechanisms such as digital signatures or certificates are easy to establish only in RR-based communications. In the PubSub paradigm, it is difficult to detect the source or the recipient of a notification, due to the decoupling between publishers and subscribers. Moreover, *event brokers* enable trusting mechanisms between publishers and *brokers* or between *brokers* and subscribers, but never directly between publishers and subscribers. Thus, a publisher is not able to detect if the recipients of a notification can be trusted, while subscribers are not able to detect if a notification has been sent from a trusted source. Overcoming this weakness involves considering additional complex trusting mechanisms that decrease efficiency [19].
- **Timeliness.** Real-time applications require controlling the timeliness of delivered messages. In PubSub-based communications it is not even possible to establish if a notification will ever be received, (see *Reliable Delivery*), thus making it impossible to delimit the time of a notification delivery from the point of view of a publisher (*i.e.*, *event brokers* can be implemented to guarantee timeliness). Additionally, if there is more than one subscriber, then the delivery time and order will depend on the specific implementation of the *event broker*, which could vary delivery times even between consecutive notifications received by the same subscribers. Therefore, timeliness cannot be enforced for publishers in PubSub-based communications [2]. In this way, the RR paradigm is required.

As a consequence of this analysis, this research work proposes the integration of the PubSub and the RR paradigms, which will contribute to seamlessly take advantage of the semantics of both paradigms and the quality properties that each of them promotes.

## 3. A Communication Model to Integrate the PubSub and the RR Paradigms

**Figure 1.**
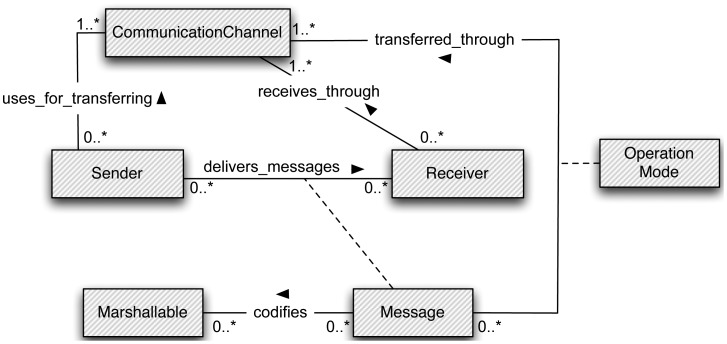
Common model for the PubSub and RR communication paradigms.

The PubSub and the RR paradigms promote different quality properties (efficiency, mobility support, adaptability, reliable delivery, security and timeliness) associated with basic communication functionalities. In this section, we propose a communication model to seamlessly integrate both paradigms in order to decrease the complexity of dealing with communication details throughout the development of ubiquitous systems. This model is depicted in Figure 1. The definition of the identified elements and a brief description of their relationships are shown in Table 2. This model will be specialized and explained in subsequent sections.

The model is inspired by the general message passing strategy for distributed systems, defined in [20]: “to initiate a communication, a process sends a message to a channel; another process acquires the message by receiving from the channel. Sending a message can be synchronous (blocking) or asynchronous (nonblocking)”. In the proposed model, a sender delivers messages to one or more receivers (*i.e.*, in the previously given definition of message passing the number of receivers is not established). Each message is delivered through a communication channel (Wi-Fi, BlueTooth, *etc.*) and with a specific operation mode (synchronous or asynchronous).

Additionally, in distributed systems, it is a general practice to transform the memory representation of the delivered data to a specific codification that is more suitable to be transferred between different processes [21–23]. The codification (also known as “marshalling” or “serialization”) depends on the specifications of the communication protocol. The proposed model takes into account that exchanged data should be “marshallable” in order to be transferred in a message between a sender and a receiver.

Starting from this simple and general communication model, which distinguishes between general concepts associated with communications (sender, receiver, message and operation mode) and those other concepts more dependent on specific technologies (communication channels and marshalling), the developers will be aware of communication semantics rather than specific communication mechanisms. Developers will be able to communicate messages to a *hybrid broker* (see Section 3.3) that will send them to the appropriate receivers through an automatically selected delivering mechanism (*i.e.*, a mechanism based either on the RR or PubSub paradigms). The mechanism selection is done on the basis of a set of attributes associated with each delivered message:

- **Operation Mode.** If a message has to be delivered asynchronously, then PubSub mechanisms are used. Otherwise, RR mechanisms are chosen.
- **Number of Receivers.** RR is used if there is only one receiver.
- **Type of Message.** The type of a message determines the mechanisms required to deliver it. For example, if a message contains an event, then it should be notified using a PubSub mechanism.

It is important to note that at least one attribute needs to be associated with each message to be delivered. The attributes associated with each message can be conflictive, for example, it might be established that a message needs to be asynchronously delivered to only one receiver and that the type of message is an event. The problem is solved by applying a priority to each attribute. In this regards, the operation mode has the top-most priority and the type of message the bottom-least one. Thus, if a conflict appears, then the operation mode guides the selection of the paradigm. In case the operation mode is not specified, then the number of receivers determines which paradigm has to be used. Finally, if neither the operation mode nor the number of receivers is set, then the type of message specifies which paradigm will be used.

The elements of the proposed model have to be specialized for each communication paradigm considered for managing the required information associated with either RR or PubSub semantics. These specializations are described in detail in the following subsections.

### 3.1. Specializing the Model to Support RR Semantics

**Figure 2.**
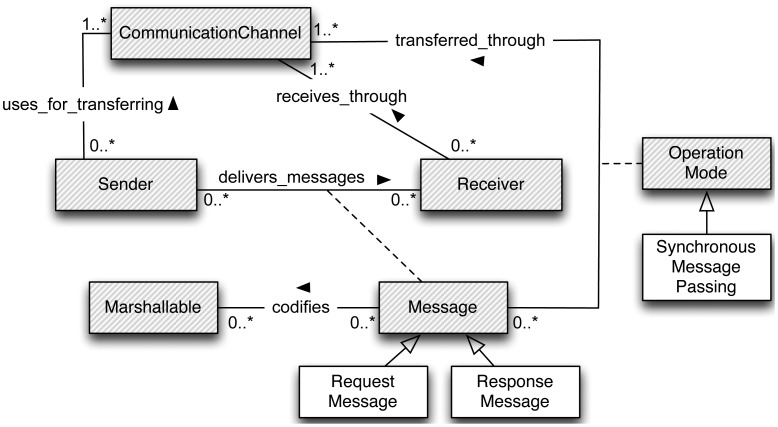
Specialized model to support RR semantics.

In RR, senders deliver messages to one specific receiver. Messages have to be marshalled according to a communication protocol and transferred by making use of a communication channel (Wi-Fi, BlueTooth, *etc.*). Messages can be intended to request information or to provide a response to a previous request. Hence, a specialization of the communication model in Figure 2 has been proposed to support RR semantics. The specialized elements of this model are defined in Table 3.

Note that an instance of a *sender* will communicate with instances of *receivers* through *messages* in order to make requests and to subsequently receive their corresponding responses. Whenever a request is received, the action specified by the instance of *Synchronous Method Invocation* element will be executed, producing a *response message*. An interesting aspect of the specialized model to support RR semantics is that it is very similar to the communication model, which highlights the simplicity of the RR paradigm.

### 3.2. Specializing the Model to Support PubSub Semantics

**Figure 3.**
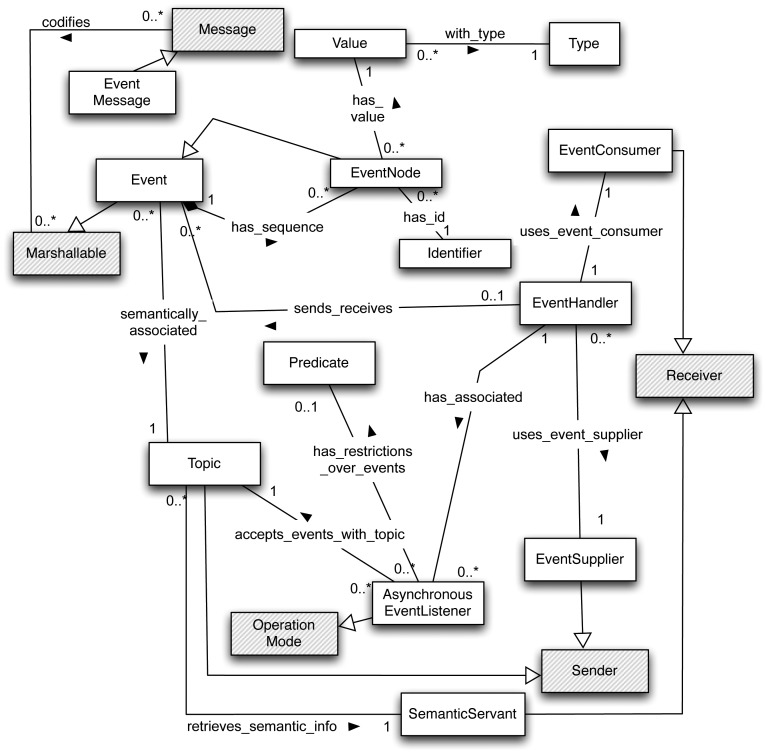
Specialized model to support PubSub semantics.

The PubSub paradigm is mainly used for notifying changes in the internal state of a sender (publisher) to a set of interested receivers (subscribers). For example, in a home automation monitoring system, if a door is opened, then an event should notify this occurrence to the applications, enabling the possibility of reflecting this state change in their corresponding user interfaces. In Figure 3, the specialized model devised to support PubSub semantics is depicted using a UML class diagram. Note that in Figure 3, the *Communication Channel* element and *delivers_message, uses_for_transferring, transferred_through* and *receives_through* relationships have been removed so as to simplify the diagram. In Table 4 the specialized elements of this model are defined.

This model is intended to support event distribution using the PubSub paradigm and its corresponding mechanisms, irrespective of the technology used to implement it. The model is proposed on the basis of different event notification models: CORBA (*i.e.*, its Notification Service) [21], OMG Data Distribution Service (DDS) [22] and ICE (using IceStorm service) [23]. In order to support most of the currently existing *event brokers, events* are represented as a collection of *event nodes*, each one being associated with an identifier and a value (with an associated type such as integer, float, string, *etc.*). However, some existing PubSub *brokers*, such as those that implement the DDS specification, also associate semantic information with events. In the proposed model, semantic information is associated with events through *topics*. More specific details about the mechanisms associated with this event model and their benefits are described in [24]. In some existing PubSub middleware solutions, semantic information is stored in a shared repository by a centralized service, which could be modeled through the *SemanticServant*. This way to associate semantic information with events also allows “topic-based” subscriptions.

The *Asynchronous EventListener* establishes the asynchronous operation mode of the PubSub paradigm. These listeners model the actions to be executed whenever an event that is related to a specific topic, and that matches a specific predicate, is received. Predicates are a way of establishing “content-based” subscriptions. For example, it is possible to establish that an event will only be received if a specific value of one of its nodes is equal to, less than or greater than another one. Depending on the communication technologies or protocols and the requirements of the ubiquitous system developed, centralized and/or decentralized publishing settings may be required (that is, connection-oriented protocols do not support broadcasts, and also broadcasts are more efficient for distributing a message to several recipients). Finally, the *EventHandler* is the mechanism that connects suppliers to consumers (*i.e.*, sender with receivers), managing event publication and subscription, as well as delivering the received event to the appropriate event listeners associated with each subscriber.

It is important to note that the PubSub model could be considered as a specialization of the RR model. At implementation level, a PubSub-based middleware could be based on a RR middleware by applying a design pattern. In fact, very prominent PubSub implementations, like [25] (centralized approach) or [26] (distributed implementation), are based on RR paradigm message passing. However, the power of the PubSub paradigm lies in that it provides higher abstraction mechanisms to deal with one-to-many, asynchronous communications for which efficient implementations can be developed [9].

### 3.3. Implementation

In distributed systems, a *broker* is a key element for connecting entities (normally, clients with servers) using a common communication protocol (GIOP in CORBA or IceP in ICE, for instance). The main idea behind supporting the implementation of the communication model is to include a higher-level *hybrid broker* model that abstracts other more specific *brokers*, and subsequently to hide whether an information exchange is made based on RR or PubSub mechanisms.

**Figure 4.**
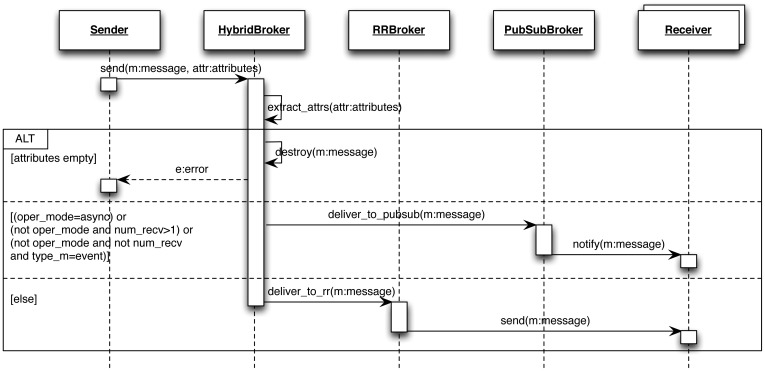
Run-time operation of the proposed *hybrid broker* as a UML sequence diagram.

The behavior model of the *hybrid broker* is depicted in Figure 4 as a UML sequence diagram and can be summarized as follows:

1. The *hybrid broker* receives a message from a source entity (for instance, an application).
2. The attributes associated with the message (operation mode, number of recipients and type of message) and provided by the developer are analyzed by the *hybrid broker*.
3. The *hybrid broker* connects to a specific broker (*i.e.*, either the RR-based or the PubSub-based broker) and sends the message to it.
4. The broker that receives the message sends it to the appropriate receiver/s.

**Figure 5.**
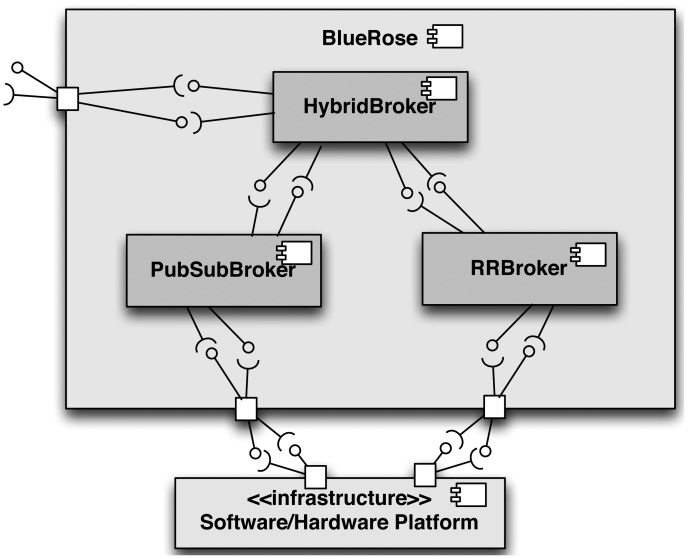
Simplified software architecture of BlueRose.

Currently, there is an implementation of the *hybrid broker* in several programming languages (*i.e.*, sharing the same design and API, but with different source codes): Java, C++, C#, Objective-C and Python. This implementation is included as part of BlueRose [27], an open source middleware that implements both the RR and the PubSub specializations with IceP and HTTP protocols, respectively. The simplified software architecture of BlueRose is shown in Figure 5.

The aim of the component-based architecture of BlueRose middleware solution is to support run-time switching between the different implementations of the specialized brokers (*PubSubBroker* and *RRBroker*), which may be based on other middleware solutions (DDS, CORBA, RMI, *etc.*) in order to take into consideration the required quality properties. The current implementation of the *hybrid broker* makes use of an XML file in order to: (1) load the appropriate implementations of the specialized brokers; (2) initialize them, *i.e.*, some middleware solutions, like CORBA, require initialization parameters.

**Figure 6.**
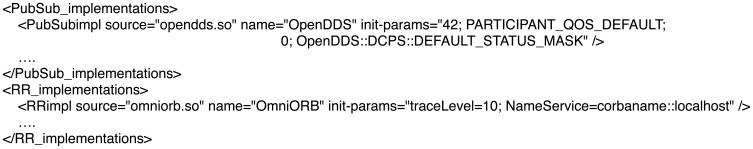
Simplified schema of the XML file used to support multiple implementations of the specialized brokers in BlueRose.

A sample schema of this file is depicted in Figure 6. Currently, the developers must manually choose the specific implementations of the components during the development of the applications. In fact, BlueRose provides an API that allows using the different the implementations of the brokers. However, a method will be incorporated to BlueRose in order to automatically select the appropriate implementations at run-time on the basis of quality properties required [28].

Using the proposed communication models in BlueRose middleware makes developers to rely on the *hybrid broker* instead of using specific brokers for different communication functionalities. This contributes to separate the software (services and applications) from specific implementations of the RR or PubSub paradigms, which is accomplished by using particular communication channels and protocols. Therefore, if any of the communication requirements change in the future, it will not be necessary to modify the implementation of the supported software services and applications, thus improving software maintainability.

## 4. An Example: Managing a Home Automation System

The proposal has been applied to the development of a real home control system for elderlies and people with cognitive disabilities (autism, dysphasia, cerebral palsy, *etc.*). The home is equipped with a series of sensors and actuators (*i.e.*, doors, blinds, light, *etc.*). The system offers two main functionalities:

- **Control**. The users can control the environment using mobile devices, for instance, they can switch lights on and off, open and close doors and blinds, *etc.* This could help to strengthen user independence.
- **Remote monitoring**. A caregiver could monitor the activities of the user to help in case unsafe situations happen. For example, if the user leaves the house (*i.e.*, door opened), then an event will notify the caregiver in order to react accordingly.

**Figure 7.**
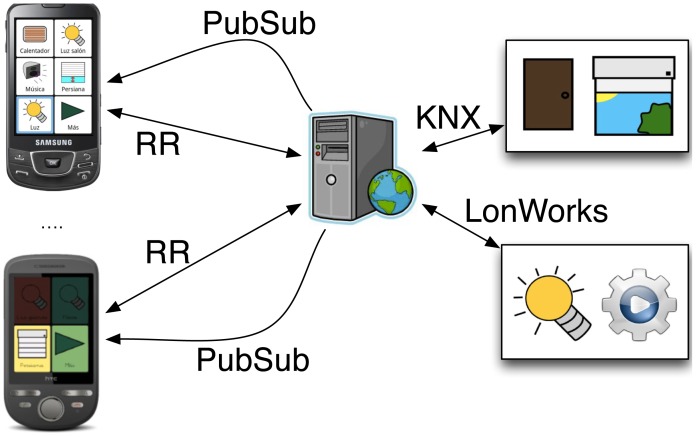
Architecture of the home automation system.

**Figure 8.**
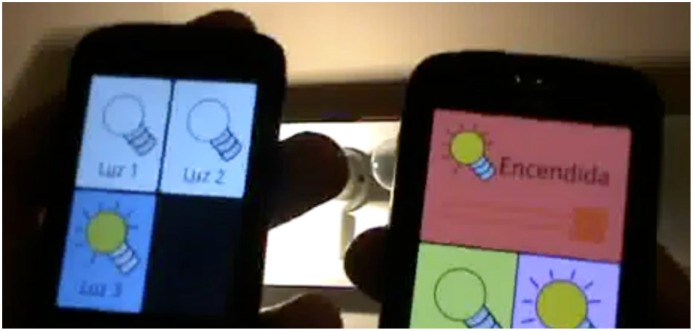
Two users interacting with the home control environment at the same time.

In this system, a mobile application interacts with domotic devices (sensors and actuators) from different manufacturers and based on two specifications: KNX and LonWorks. The system architecture for the home automation system is depicted in Figure 7. In this architecture, a service enables uniform control of heterogeneous home automation devices. The specific communication protocols that are used to exchange information with the domotic devices (*i.e.*, KNX or LonWorks) only permit message-passing communications, in particular, by making use of the RR paradigm. However, it is possible to incorporate PubSub mechanisms in the service in order to notify the different instances of the mobile application about a change in the state of any of the devices, thereby enabling remote monitoring. Figure 8 shows a snapshot of two users interacting with a home control environment at the same time. One of users could be a patient and the other one a caregiver supervising how the patient interacts with the physical environment.

A possible approach to integrate PubSub-based and RR-based technologies and paradigms is to directly connect the mobile application and the service to a set of *brokers*. However, this idea presents two drawbacks:

- Development complexity is high, since developers need to have a deep knowledge about the mechanisms and technologies they are using in order to implement applications and services with several *brokers*.
- If new communication technologies are to be incorporated in order to fulfill additional requirements, then several parts of the mobile application and the service must be recoded.

The models presented in this paper have been applied to the development of the described home automation system. The aim is to overcome these technical issues as follows. Developers make use of the *hybrid broker* to abstract the complexity of selecting the specific broker that will be used to communicate the information, which in turn, hides the internal communication mechanism to be used. For example, the *hybrid broker* hides different communication channels, protocols and middleware technologies associated with them. Likewise, the implementation of the *hybrid broker* is also based on the communication model, which clearly separates general concepts associated with communications (sender, receiver, message and operation mode) from those specific ones more related to technologies (communication channel and marshalling). Thus, it is possible to take into consideration new communication technologies without involving the re-implementation of either the mobile application or the service, and without modifying the *hybrid broker*, except for its associated middleware technologies.

The BlueRose middleware supports the implementation of the system by means of the *hybrid broker*. The specialized broker for PubSub-based communications has been implemented on the basis of ICE middleware, integrating this implementation into BlueRose as a PubSub component. The same was achieved by using RR-based communications and IceP, KNX and LonWorks technologies, each of them supported by a different implementation of the RR component of BlueRose. The service that enables the interaction between the mobile devices and the home automation devices has been implemented to load the different versions of the RR component depending on the communication participants: a mobile device using the IceP communication protocol, a KNX-based device, or a LonWorks-based device.

For example, to interact with a KNX device, a mobile device sends an IceP message to the service, which extracts its contents and resends the message through KNX protocol. Note that, in order to avoid mapping from different message specifications, all the messages enclose information that enables the service to create new messages or notifications according to the specific technology specification. Thus, the messages that are sent by the mobile application contain an identifier of the receiver, the operation (to read or to write), the data type (*i.e.*, KNX and LonWorks specifications use specific data types, but in the developed system, abstract data types have been proposed in order to avoid interoperability issues between both specifications) and optionally the enclosed information (when the operation consists of writing a data in a domotic device). The enclosed information is marshalled according to the protocol that is used to send the message (in this case IceP). The service decodes this information on the basis of the given data type and re-marshalls it according to the required protocol (KNX or LonWorks).

The advantage of using the proposed *hybrid broker* is that developers only have to decide the specialized communication model that they want to use (*i.e.*, either the RR or the PubSub specialization). For example, when an event should be delivered asynchronously and to several recipients, then a PubSub specialization must be selected by the *hybrid broker*. Conversely, the messages exchanged between the mobile application and the service should be synchronous and delivered to one receiver only, thereby selecting the RR specialization. Moreover, the *hybrid broker* hides the specific communication mechanisms (including protocols and technologies) to the developers, making it easier to implement the system. Furthermore, it facilitates to meet future specifications (new devices from other manufacturers and incorporating new middleware technologies), since new implementations of the PubSub and the RR components can be easily incorporated to BlueRose, as described in Section 3.3.

The mobile application has been developed for the Android mobile platform, whereas the service can be deployed in any Java-compatible operating system (Windows, Linux, MacOSX, *etc.*). More details of the home automation system (also known as Kora) can be found in [29]. The Java source code is available in [30].

## 5. Related Work

Several works have dealt with the communication between software entities in ubiquitous systems through the RR or the PubSub paradigms. These approaches are mainly focused on overcoming their weaknesses (see Section 2). However, a small number of research works have previously addressed the problem of decreasing the complexity of developing integrated ubiquitous systems based on heterogeneous communication schemes, technologies, architectures and paradigms.

In [4], the modification of traditional Sun implementation of the RPC paradigm is proposed in order to incorporate mechanisms to provide support to mobility. However, in that proposal, it is not clear how that paradigm can be improved to efficiently deliver information to several receivers. Saif *et al.* [3] propose to promote the usage of event-based communications (apparently through the PubSub paradigm), but the authors do not clarify which specific mechanisms should be used to fulfill reliable delivery, timeliness and/or security requirements. Corsaro *et al.* [2] analyze these weaknesses in the PubSub paradigm and propose the incorporation of several mechanisms to avoid them. However, in that work, it is pointed out that these problems remain unsolved. OMG DDS [22] is a standard specification of a PubSub-based middleware intended to support real-time, reliable communications through the specification of application-level QoS parameters. Nonetheless, security is not addressed in the standard [31], it does not support interaction between heterogeneous participants [32], and it does not scale very well in wide area networks (WANs) [33].

The proposal presented in this research work integrates the PubSub and the RR paradigms in order to accomplish some properties that are not easily fulfilled with only PubSub-based approaches. For example, since the proposed models are independent from specific communication technologies or implementations, it could serve as a basis to solve interoperability issues with heterogeneous participants or to take into consideration specific requirements, like scalability or security, through specific design decisions and implementations of the *brokers*.

Message-oriented middleware (MOM) solutions provide an intermediary broker to deliver messages in an asynchronous and decoupled manner. There are two main categories of MOM technologies [34]: PubSub and message queueing. In ubiquitous systems, both categories are relevant since they promote mobility support [35]. However, Happe *et al.* [36] have analyzed the influence of the inclusion of MOM in software architecture models and they have detected that these technologies require additional efforts as well as detailed knowledge of the used infrastructure, which leads to erroneous assumptions or uses of MOM technologies. In our proposal, existing MOM solutions can be used to implement the specialized PubSub model, with the main advantage of decreasing the efforts to deal with the peculiarities of these technologies.

Several authors have highlighted the importance of combining both asynchronous and synchronous communications in mobile environments (AmI systems or mobile collaborative systems, for instance). Rodríguez-Covili *et al.* [37] propose a reference architecture for mobile shared workspaces in which a “communication component” supporting both interaction modes is introduced. The proposal that we have presented herein can be considered as a technical and as a conceptual complement to the design proposal described in that work. In [38] it is recommended to make use of notifications and reliable connections in *ad-hoc* networks that support mobile collaborative applications, which leads to making use of both the RR and PubSub communication paradigms (although this is not explicitly mentioned). Bamis *et al.* [39] propose a framework for behavior interpretation of elders in AmI systems. This framework poses the need of using asynchronous event notifications and synchronous communications to interact with centralized services. SeDiM [40] is a middleware framework to allow heterogeneous service discovery protocols to interoperate. The work presented herein complements this work by proposing models to support message exchange and event distribution between heterogeneous applications or services.

In summary, the presented proposal intends to provide the integration of the RR and the PubSub paradigms in order to enable easy development of software solutions that require a combination of synchronous and asynchronous communications. In contrast with other solutions specifically developed for ubiquitous systems, our proposal aims to be more technology-independent and to offer a higher abstraction level than message delivery. Moreover, the proposed model aims to facilitate the integration and easier incorporation of heterogeneous technologies in ubiquitous systems, which provides clear benefits, as highlighted in recent contributions by other authors [41,42].

## 6. Conclusions and Future Work

In this paper a communication model to seamlessly integrate the RR and the PubSub paradigms has been presented. The aim is to facilitate the fulfillment of several functional and non-functional requirements associated with information communication in ubiquitous systems.

At the functional level, the communication model and its implementation as a *hybrid broker* enable to synchronously exchange messages and to deliver asynchronous notifications. Developers only require to be aware of communication semantics rather than specific communication mechanisms. For example, these two communications paradigms are adequate for developing several kinds of software applications and services, such as chats, monitoring of users' tasks, exchange of multimedia information and real-time collaboration, which are frequently demanded functionalities in ubiquitous systems. However, currently, the development of software applications require the usage of several middleware solutions, each of them with different underlying designs and implementations, thereby usually forcing developers to focus on using distinct technologies rather than on actually providing the expected functionalities in a more independent way.

As far as quality properties are concerned, the combination of the RR and the PubSub paradigms allows software developers to flexibly switch between these two paradigms when building software applications and services for efficient, mobile, reliable, adaptable, secure and timely ubiquitous systems. Furthermore, the model presented in this work, and the *hybrid broker* supporting its implementation, contribute to ease the development of this kind of systems. It is important to note that maintainability, reusability and extensibility properties are also improved as a result of the separation between conceptual and technical issues, and between the high-level software (applications and services) and the actual implementations of the RR and the PubSub specializations of the communication model. Moreover, the communication channels and protocols associated with the implementation of the specialized elements of the RR and the PubSub models can be changed without modifying the implementation of the *hybrid broker*. The fulfillment of reusability and extensibility properties is considered one of the main challenges in the development of AmI systems [43]. It is worth to be mentioned that the goal of the proposal presented herein is not to guarantee the fulfillment of quality properties, but to take them into consideration through the integration of specific implementations of PubSub-based and RR-based middleware solutions.

The benefits of the proposal presented herein have been highlighted in an example consisting of a home automation system, including a mobile application, a service and a set of home automation devices. The implementation of the *hybrid broker* is actually part of BlueRose, a middleware for ubiquitous systems that implements both the RR and the PubSub specializations with specific communication technologies.

As future work, new abstractions will be incorporated to the models supporting the RR and PubSub paradigms in order to support the adjustment of QoS parameters for the messages to be exchanged and to coordinate different communication participants. The proposed model, as well as the resulting specialized ones supporting the RR and the PubSub communication paradigms, should be evaluated by means of specific implementations so as to effectively measure the degree of fulfillment of the quality properties that in this contribution have been dealt with. Finally, the proposal presented herein will be part of a framework to support the development of complex ubiquitous systems supporting dynamic discovery of nearby entities (applications or services) and context management.

## Figures and Tables

**Table 1.** Quality properties promoted by the PubSub and the RR paradigms.

**Property**	**PubSub**	**RR**
Efficiency	partial	partial
Mobility Support	✓	
Adaptability	✓	
Reliable Delivery		✓
Security	partial	✓
Timeliness		✓

**Table 2.** Elements of the communication model.

**Element**	**Definition**
Receiver	A target for the reception of some information.
Sender	The deliverer of some information to one or more receivers.
Message	Information that is exchanged between a sender and a receiver (e.g., an application and a service).
Communication Channel	A communication channel (BlueTooth, Wi-Fi, *etc.*) to exchange messages.
Marshallable	Model of a coded or serialized object that can be transferred as part of a message, as specified by the communication protocol.
Operation Mode	How the messages are delivered through the communication channel: synchronously or asynchronously.
delivers message	A sender can deliver a message to several recipients. The mechanism to deliver the message depends on the operation mode.
codifies	A message contains codified data.
uses for transferring	A sender delivers a message through one or more communication channels.
transferred through	A message can be delivered to a receiver through a set of communication channels, which can be different from the channels that are used by the sender.
receives through	A message can be received from one or more communication channels, that can be shared or not with the sender.

**Table 3.** Definition of the elements of the specialized model to support RR semantics.

**Element**	**Definition**
Request Message	A message intended to request some information to a receiver.
Response Message	A message to provide a response to a previous request.
Synchronous Method Invocation	Models the action to be executed whenever messages are synchronously received.

**Table 4.** Definition of the elements of the specialized model to support PubSub semantics.

**Element**	**Description**
Event Message	Specialization of a message to model notifications.
Event	A notification of a change in the state of a publisher. A further explanation of this model is described in [24].
Event Node	An event node is the specification of a piece of information associated with an event. It is an identifier-type-value tuple.
Topic	Both events and event nodes will have an associated topic, that is, a way of specifying their semantics.
Event Consumer	Receiver of an event.
Event Supplier	Sender of an event.
Asynchronous EventListener	They execute certain actions whenever a specific event is notified to an event consumer. A listener may trigger the actions if the notified event is associated to a topic and/or if the event complies with a set of restrictions (a predicate).
Predicate	Element allowing the specification of the set of restrictions over the nodes of an event. Predicates are used by event listeners to trigger their associated actions.
Event Handler	Element that publishes events through an instance of *event supplier* and receiving published events through an instance of *event consumer*. It also delivers received events to the appropriate event listener.
Semantic Servant	Service storing the topics, so as to retrieve them.
